# Desempenho do Escore SHARPEN e do Índice de Comorbidade de Charlson para Predição de Mortalidade durante a Internação Hospitalar e após a Alta na Endocardite Infecciosa

**DOI:** 10.36660/abc.20230441

**Published:** 2024-01-19

**Authors:** Sofia Giusti Alves, Fernando Pivatto, Filippe Barcellos Filippini, Gustavo Paglioli Dannenhauer, Gabriel Seroiska, Helena Marcon Bischoff, Luiz Felipe Schmidt Birk, Diego Henrique Terra, Daniel Sganzerla, Marcelo Haertel Miglioranza

**Affiliations:** 1 Hospital de Clínicas de Porto Alegre Porto Alegre RS Brasil Hospital de Clínicas de Porto Alegre, Porto Alegre (HCPA), RS – Brasil; 2 Hospital Nossa Senhora da Conceição Porto Alegre RS Brasil Hospital Nossa Senhora da Conceição (HNSC), Porto Alegre, RS – Brasil; 3 Instituto de Cardiologia de Santa Catarina São José SC Brasil Instituto de Cardiologia de Santa Catarina, São José, SC – Brasil; 4 Universidade Federal de Ciências da Saúde de Porto Alegre Porto Alegre RS Brasil Universidade Federal de Ciências da Saúde de Porto Alegre (UFCSPA), Porto Alegre, RS – Brasil; 5 Unimed Porto Alegre Cooperativa Médica Porto Alegre RS Brasil Unimed Porto Alegre Cooperativa Médica, Porto Alegre, RS – Brasil; 6 Instituto de Cardiologia do Rio Grande do Sul Laboratório de Pesquisa e Inovação em Imagem Cardiovascular Porto Alegre RS Brasil Instituto de Cardiologia do Rio Grande do Sul (ICFUC-RS) – Laboratório de Pesquisa e Inovação em Imagem Cardiovascular, Porto Alegre, RS – Brasil; 7 Hospital Mãe de Deus Porto Alegre RS Brasil Hospital Mãe de Deus, Porto Alegre, RS – Brasil

**Keywords:** Endocardite, Comorbidade, Análise de Sobrevida

## Abstract

**Fundamento:**

O SHARPEN foi o primeiro escore desenvolvido especificamente para a predição de mortalidade hospitalar em pacientes com endocardite infecciosa (EI), independentemente da realização de cirurgia cardíaca.

**Objetivos:**

Analisar a capacidade do escore SHARPEN na predição de mortalidade hospitalar e mortalidade após a alta e compará-la à do Índice de Comorbidade de Charlson (ICC).

**Métodos:**

Estudo retrospectivo do tipo coorte incluindo internações por EI (segundo os critérios de Duke modificados) entre 2000 e 2016. A área sob a curva ROC (AUC-ROC) foi calculada para avaliar a capacidade preditiva. Curvas de Kaplan-Meier e regressão de Cox foram realizadas. Um valor de p < 0,05 foi considerado estatisticamente significativo.

**Resultados:**

Estudamos 179 internações hospitalares. A mortalidade hospitalar foi 22,3%; 68 (38,0%) foram submetidos à cirurgia cardíaca. Os escores SHARPEN e ICC (mediana e intervalo interquartil) foram, respectivamente, 9(7-11) e 3(2-6). O escore SHARPEN mostrou melhor predição de mortalidade hospitalar em comparação ao ICC nos pacientes não operados (AUC-ROC 0,77 vs. 0,62, p = 0,003); não foi observada diferença no grupo total (p=0,26) ou nos pacientes operados (p=0,41). Escore SHARPEN >10 na admissão foi associado a uma menor sobrevida hospitalar no grupo total (HR 3,87; p < 0,001), nos pacientes não operados (HR 3,46; p = 0,006) e de pacientes operados (HR 6,86; p < 0,001) patients. ICC > 3 na admissão foi associada a pior sobrevida hospitalar nos grupos total (HR 3,0; p = 0,002), de pacientes operados (HR 5,57; p = 0,005), mas não nos pacientes não operados (HR 2,13; p = 0,119). A sobrevida após a alta foi pior nos pacientes com SHARPEN > 10 (HR 3,11; p < 0,001) e ICC > 3 (HR 2,63; p < 0,001) na internação; contudo, não houve diferença na capacidade preditiva entre esses grupos.

**Conclusão:**

O SHARPEN escore foi superior ao ICC na predição de mortalidade hospitalar nos pacientes não operados. Não houve diferença entre os escores quanto à mortalidade após a alta.

## Graphical abstract

**Figura Central:**
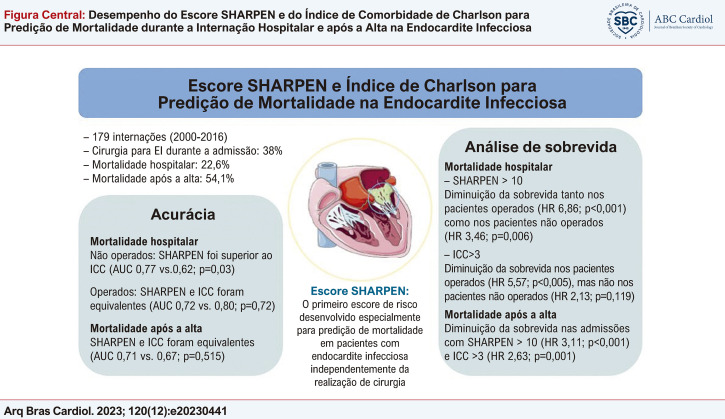
: Desempenho do Escore SHARPEN e do Índice de Comorbidade de Charlson para Predição de Mortalidade durante a Internação Hospitalar e após a Alta na Endocardite Infecciosa

## Introdução

A endocardite infecciosa (EI) tem uma alta incidência, com 1,5-11,6 casos por 100 000 pessoas,^1^ e taxas de mortalidade hospitalar que variam entre 17,5% e 30%.^1-3^ Pacientes que sobrevivem ao primeiro episódio de EI continuam apresentando mortalidade e morbidade elevadas, principalmente no primeiro ano de alta.^4^ Mudanças recentes no perfil epidemiológico da EI podem haver contribuído para a manutenção da morbidade e da mortalidade elevada. A incidência de EI tem aumentado em pacientes com fatores de risco para eventos adversos, tais como idade avançada, comorbidades, próteses valvares, e dispositivos cardíacos; além disso, um aumento de casos de endocardite nosocomial tem sido relatado.^2,5,6^

Considerando o impacto da EI na saúde, a otimização de sua avaliação e de seu tratamento é essencial. Há evidência de melhora nos desfechos clínicos com o uso de um uma estratégia de alarme multidisciplinar e protocolos padronizados de EI com base na gravidade da doença.^7-10^ Nesse contexto, a estratificação de risco baseada em um escore pode auxiliar no encaminhamento de pacientes de alto risco para centros especializados ou de terapia intensiva.^11^ Considerando que a cirurgia precoce está associada a menor mortalidade por EI, os escores de risco podem ser úteis para direcionar a seleção dos pacientes.^12^ Ainda, sua aplicação para predição da mortalidade após internação por EI pode ajudar a identificar os pacientes que se beneficiariam de um acompanhamento mais próximo após a alta.

Vários escores de risco cirúrgicos foram desenvolvidos especificamente para EI^13-20^ e comparados com escores de risco cirúrgicos tradicionais, como o EuroSCORE e o escore STS.^21-23^ No entanto, esses escores não foram validados em pacientes não operados, que representam quase 50% de todas as internações por EI^6^, ou na avaliação prognóstica em longo prazo. O estudo *The ICE-Prospective Cohort Study* é o único que avaliou a mortalidade por EI após a alta, a qual foi determinada aos seis meses.^24^ Assim, é necessário melhorar a avaliação prognóstica dos pacientes sob tratamento clínico.

SHARPEN é um escore de risco para EI desenvolvido por Chee et al.^11^ para predizer mortalidade hospitalar em pacientes operados e não operados.^25^ Nosso estudo tem como objetivo avaliar o valor prognóstico do escore SHARPEN durante a internação por EI tanto em pacientes operados como não operados, além de comparar seu desempenho com o Índice de Comorbidade de Charlson (ICC).^26^

## Pacientes e métodos

Realizamos um estudo retrospectivo unicêntrico do tipo coorte incluindo todos os casos de EI ativa (em tratamento com antibiótico)^27^ entre 2000 e 2016 em pacientes com idade ≥ 18 anos. Foram incluídos somente os pacientes com diagnóstico definitivo de EI de acordo com os critérios de Duke.^28^ Nossa instituição é um hospital escola público terciário localizado no sul do Brasil. O hospital tem 784 leitos e o acesso à saúde é concedido exclusivamente pelo Sistema Único de Saúde, principalmente para pacientes de baixa renda. Uma média de 30-60 substituições de válvula são realizadas por ano na instituição. O estudo foi aprovado pelo comitê de ética em pesquisa.

**Figura 1:**
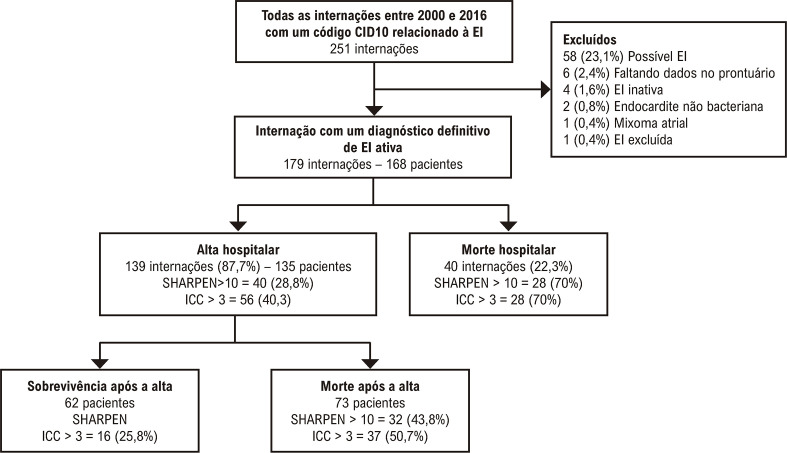
– Fluxograma do estudo; EI: Endocardite Infecciosa; CID: Classificação Internacional de Doenças; ICC: Índice de comorbidade de Charlson; *critério modificado de Duke; dados apresentados como n (%).

Internações por EI foram identificadas pelo código da Classificação Internacional de Doenças, 10ª revisão (CID-10)^29^ registrado no resumo da alta ou em qualquer outro momento durante a internação. Os seguintes códigos foram pesquisados: B37.6 (Endocardite por Candida), I33.0 (Endocardite aguda e subaguda), I33.9 (Endocardite aguda e subaguda não especificada), I38 (Endocardite de valva não especificada), e I39.8 (Endocardite e transtornos valvulares cardíacos em doenças classificadas em outra parte). Após esse rastreamento inicial, os prontuários dos pacientes foram revisados para assegurar que os critérios de inclusão foram preenchidos. A Figura 1 apresenta o fluxograma do estudo. Dados do período de internação foram coletados dos prontuários médicos físicos e eletrônicos. A avaliação no seguimento pós-alta envolveu a avaliação dos prontuários médicos para verificar se os pacientes sobreviventes tiveram consulta e/ou internações após a alta, número de telefone dos demais pacientes e, finalmente, revisão dos atestados de óbito. Todos os pacientes que não foram registrados como falecidos até em nenhuma dessas fontes de dados até 1º de outubro de 2022 (que marcou a conclusão da avaliação do seguimento) foram considerados vivos.

O escore SHARPEN^11^ (2-20 pontos, material suplementar S1) foi calculado retrospectivamente para cada internação e foi realizada classificação como baixo ou alto risco para mortalidade hospitalar de acordo com o melhor ponto de corte observado. Foram atribuídos três pontos para pressão sistólica < 90mmHg ou usuário de drogas não injetáveis; dois pontos para manifestações de insuficiência cardíaca (IC) durante internação, creatinina na admissão > 2,26mg/dL, diagnóstico de pneumonia nosocomial, pico de proteína C reativa (PCR) > 200mg/dL durante a internação; e 2, 4, e 6 pontos para os grupos de < 50, 50-65, e > 65 anos, respectivamente). O diagnóstico de IC foi definido com base nos critérios de Framingham.^30^ Pneumonia nosocomial foi definida como pneumonia ocorrendo ≥ 48 horas após a admissão hospitalar. O ICC foi calculado para cada paciente (Material Suplementar S2) e as definições de comorbidades do estudo original foram usados.^26^ A urgência da operação foi definida de acordo com os critérios EuroSCOREII.^27^

### Análise estatística

Os dados foram analisados usando o programa SPSS, versão 21.0, e MedCalc, versão 12.5. Para análise descritiva, as variáveis categóricas foram expressas em frequências absolutas e relativas, as variáveis contínuas com distribuição normal em média [± desvio padrão (DP)], e as variáveis contínuas sem distribuição normal em mediana [Intervalo Interquartil (IIQ)]. O teste de Shapiro-Wilk foi usado para testar a normalidade da distribuição. Para comparações entre os grupos, as variáveis categóricas e as variáveis quantitativas com distribuição normal foram comparadas pelo teste do qui-quadrado e pelo teste t de Student, respectivamente. O teste exato de Fisher foi usado em caso de baixa frequência dos dados. A capacidade preditiva do escore SHARPEN e do ICC foi avaliada calculando-se a Área sob a Curva ROC (AUC-ROC). As AUC-ROCs foram comparadas usando o teste DeLong, o melhor ponto de corte em cada sistema de escores foi determinado pelo índice de Youden. A análise de sobrevida foi realizada usando curvas de Kaplan-Meier. Modelos de regressão de Cox foram usados para calcular a razão de risco (*Hazard Ratio*, HR) da mortalidade hospitalar e após a alta. Valores de p<0,05 foram considerados estatisticamente significativos.

## Resultados

### Características basais da amostra

Das 251 internações inicialmente identificadas, 179 internações de 168 pacientes preencheram os critérios de inclusão; 10 pacientes tinham mais de uma internação por EI durante o período do estudo (Material Suplementar S3). O tempo mediano de internação foi 45 (33-64) dias, com 93 (52,0%) de internações na unidade de terapia intensiva (excluindo admissões somente para monitoramento pós-operatório). As características basais da amostra estão apresentadas na Tabela 1. A terapia com antibióticos foi iniciada 1 (0-6) dia (período mediano) após a admissão. Não houve casos de EI em dispositivos intra-cardíacos.

**Tabela 1:** – Características basais e complicações hospitalares

Variável	Total (n = 179)	Alta hospitalar (n = 139)	Morte hospitalar (n = 40)	p
***Características basais***				
Idade (anos)*	57,4 (42,3-68,5)	54,7 (40,6-66,0)	64,7 (54,5-72,1)	,004
Sexo masculino	126 (70,4)	101 (72,7)	25 (62,5)	,30
FEVE (%)	63 (58-68)	63 (58-68)	62 (56-69)	,97
EI do lado esquerdo	164 (91,6)	124 (89,2)	40 (100)	,020
Hipertensão	92 (51,4)	68 (48,9)	24 (60,0)	,29
Diabetes†	37 (20,7)	27 (19,4)	10 (25,0)	,58
Cirurgia cardíaca prévia	31 (17,3)	24 (17,3)	7 (17,5)	1,0
PAS < 90mmHg na admissão*	26 (14,5)	15 (10,9)	11 (27,5)	,018
Pico de PCR durante a internação > 200mg/L*	26 (14,5)	16 (11,5)	10 (25,0)	,060
EI de prótese valvar	23 (12,8)	18 (12,9)	5 (12,5)	1,0
Creatinina > 2,26mg/dL na admissão*	20 (11,2)	12 (8,6)	8 (20,0)	,082
DRC com necessidade de diálise †	14 (7,8)	8 (5,8)	6 (15,0)	,088
Uso de droga endovenosa*	13 (7,3)	11 (7,9)	2 (5,0)	,74
HIV	9 (5,0)	6 (4,3)	3 (7,5)	,42
Disfunção cardíaca (FEVE ≤ 40%)	8 (4,5)	6 (4,3)	2 (5,0)	,85
***Complicações hospitalares***				
Regurgitação moderada/grave	107 (59,8)	79 (56,8)	28 (70,0)	,19
Insuficiência cardíaca*†	97 (54,2)	69 (49,6)	28 (70,0)	,036
Cirurgia cardíaca	68 (38,0)	54 (38,8)	14 (35,0)	,80
Pneumonia (≥ 48h após admissão)*	38 (21,2)	22 (15,8)	16 (40,0)	,002
Eventos embólicos (excluindo eventos cerebrovasculares)	35 (19,6)	25 (18,0)	10 (25,0)	,45
Hemodiálise‡	33 (18,4)	12 (9,2)	21 (61,8)	< ,001
Complicações intracranianas (hemorragia/AVC isquêmico)	31 (17,3)	21 (15,1)	10 (25,0)	,22
Ruptura de cordas tendinosas	23 (12,8)	19 (13,7)	4 (10,0)	,73
Abscesso perivalvar	18 (10,1)	14 (10,1)	4 (10,0)	1,0
Fístula	14 (7,8)	10 (7,2)	4 (10,0)	,52
Pseudoaneurisma	3 (1,7)	3 (2,2)	0 (0)	1,0

Dados apresentados em mediana (intervalo interquartil) ou número (%); AVC: acidente vascular cerebral; DRC: doença renal crônica; HIV: vírus da imunodeficiência humana; EI: endocardite infecciosa; FEVE: fração de ejeção do ventrículo esquerdo; NS: não significativo; PAS: pressão arterial sistólica; *Componente do escore SHARPEN; †Componente do Índice de Comorbidade de Charlson. ‡Excluindo pacientes com doença renal crônica com necessidade de diálise antes da admissão hospitalar (n = 14).

Ecocardiografia transesofágica foi realizada na maioria dos pacientes (n = 145; 81,0%). Foram detectadas vegetações em 162 pacientes (90,5%), que eram maiores que 10mm em 69 (38.5%) pacientes. EI da valva aórtica (n = 68; 38,0%) ou da valva mitral (n = 60; 33,5%) foi a apresentação mais comum, e 36 (20,1%) pacientes apresentaram envolvimento em mais de uma válvula. Culturas sanguíneas positivas foram encontradas em 154 (86,0%) pacientes; *Staphylococcus aureus* (22.0%) e *Streptococcus viridans* (15,1%) foram os agentes mais comuns. A infecção associada com cateter ocorreu em 12 (6,7%) da amostra.

Embora em 87 (48,6%) das internações houvesse indicação de cirurgia, o procedimento foi realizado durante a mesma internação em somente 68 (78,2%) delas – em caráter de urgência em 64 (94,1%) e em caráter emergencial em quatro (5,9%) pacientes. As indicações cirúrgicas foram IC aguda (n = 54; 79,4%) e infecção não controlada (n = 28; 41,2%). As principais razões de não se operar durante a mesma internação apesar da indicação foram: plano de uma cirurgia eletiva em uma internação subsequente (n = 7; 6,8%) e instabilidade hemodinâmica (n = 5; 26,3%). Os procedimentos mais realizados foram o implante de válvula aórtica mecânica (n = 19, 10,6%) e o implante de válvula aórtica biológica (n = 12, 6,7%). O tempo médio (± DP) de *bypass* cardiopulmonar e isquemia foram 136 (± 46) e 104 (± 42) minutos, respectivamente.

A mortalidade hospitalar foi 22,3% [Intervalo de Confiança (IC) de 95%: 16,2-28,4%)], e o choque séptico foi a principal causa de morte (n = 20; 11,2%). A taxa de mortalidade não foi diferente entre pacientes operados e não operados (20,6% vs. 23,4%, p = 0,797).

### Avaliação prognóstica usando o escore SHARPEN e o ICC para predição de mortalidade hospitalar

Os pacientes apresentaram um escore SHARPEN mediano de 9 (7-11) pontos. Os escores SHARPEN (mediana e IIQ) dos pacientes após a alta e dos pacientes que foram a óbito durante a internação foram 9 (7-11) e 11 (9-13) pontos, respectivamente (p < 0,001). O melhor ponto de corte para predição de mortalidade no escore SHARPEN foi > 10 pontos. No geral, 111 (62,0%) internações foram classificadas como de baixo risco (2-10 pontos) e 68 (38,0%) como de alto risco (11-20 pontos), com taxas de mortalidade hospitalar entre 10,8 e 41,2%, respectivamente (p < 0,001).

Os pacientes apresentaram um ICC mediano de 3 (2-6) pontos. Os escores ICC (mediana e IIQ) dos pacientes após a alta e dos pacientes que foram a óbito durante a internação foram 3 (1-5) e 5 (3-7), respectivamente (p < 0,001). O melhor ponto de corte para predição de mortalidade para o ICC foi >3 pontos. No geral, 95 (56,1%) internações foram classificadas como de baixo risco (≤3 pontos) e 84 (46,9%) como de alto risco (>3 pontos), com taxas de mortalidade hospitalar entre 12,5 e 33,3%, respectivamente (p < 0,001). A Tabela 2 apresenta as características do escore SHARPEN e do ICC em todos os pacientes, nos pacientes operados e nos pacientes não operados.

**Tabela 2:** – Características do escore SHARPEN e do Índice de Comorbidade de Charlson

Estatísticas % (IC95%)	SHARPEN > 10			ICC > 3		
	Total	Não operados	Operados	Total	Não operados	Operados
Sensibilidade	70,0 (53,5-83,4)	71,4 (41,9-91,1)	69,2 (48,2-85,7)	70,0 (53,5-83,4)	69,2 (48,2-85,7)	71,4 (41,9-91,6)
Especificidade	71,2 (62,9-78,6)	72,2 (58,3-83,5)	70,6 (59,7-79,9)	59,7 (51,1-67,9)	50,6 (39,5-61,6)	74,1 (60,4-85)
Razão de verossimilhança positiva	2,43 (1,75-3,39)	2,57 (1,49-4,43)	2,35 (1,55-3,57)	1,74 (1,30-2,31)	1,40 (1,0-1,96)	2,76 (1,57-4,82)
Razão de verossimilhança negativa	0,42 (0,26-0,68)	0,40 (0,17-0,92)	0,44 (0,24-0,79)	0,50 (0,31-0,82)	0,61 (0,33-1,12)	0,39 (0,17-0,90)
Mortalidade	22,3	20,6	23,4	22,3	23,4	20,6
Valor preditivo positivo	41,1 (33,4-49,3)	40,0 (27,9-53,4)	41,8 (32,1-52,2)	33,3 (27,2-39,9)	30 (23,5-37,5)	41,67 (28,9-55,5)
Valor preditivo negativo	89,2 (83,6-93,1)	90,7 (80,7-95,8)	88,2 (80,6-93,1)	87,4 (80,9-91,9)	84,3 (74,4-90,9)	90,9 (81,1-95,9)
Acurácia	70,9 (63,7-77,5)	72,1 (59,8-82,3)	70,3 (60,8-78,6)	62,0 (54,5-69,1)	54,95 (45,2-64,4)	73,5 (61,4-83,5)

*ICC: índice de comorbidade de Charlson; IC: intervalo de confiança.*

**Figura 2:**
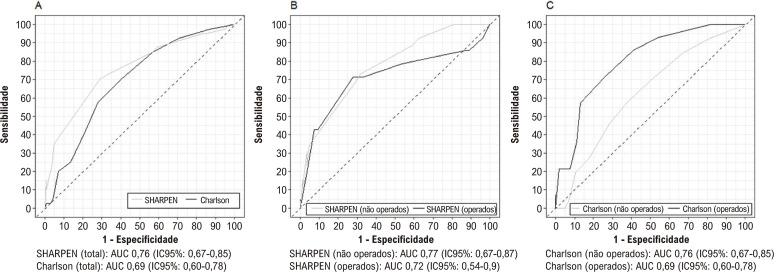
– Curvas ROC do escore SHARPEN e do Índice de Comorbidade de Charlson para a predição de mortalidade hospitalar. 2A: Curvas ROC curves para o escore SHARPEN e Índice de Comorbidade de Charlson em toda a amostra; 2B: Curvas ROC para o escore SHARPEN em pacientes operados e não operados; 2C: Curvas ROC para Índice de Comorbidade de Charlson em pacientes operados e não operados, AUC: área sub a curva; IC: intervalo de confiança.

A Figura 2 apresenta as curvas ROC para predição de mortalidade hospitalar de acordo com o escore SHARPEN e o ICC. Não houve diferença na AUC do escore SHARPEN entre os pacientes operados e não operados (p = 0,058). Por outro lado, encontramos uma diferença estatisticamente significativa na AUC do ICC entre os pacientes operados e não operados (p = 0,039). Quando comparamos a capacidade do SHARPEN e do ICC em predizer a mortalidade hospitalar, não encontramos diferença na amostra total (p = 0,26) ou nos pacientes operados (p = 0,41). No entanto, no subgrupo de pacientes não operados, o escore SHARPEN foi superior ao ICC (p = 0,003).

### Análise de sobrevida hospitalar

Observou-se uma associação estatisticamente significativa entre o escore SHARPEN > 10 e uma sobrevida hospitalar menor, o que se manteve na análise dos pacientes operados e não operados separadamente. As curvas de sobrevida hospitalar de acordo com o escore SHARPEN são apresentadas na Figura 2.

**Figura 4:**
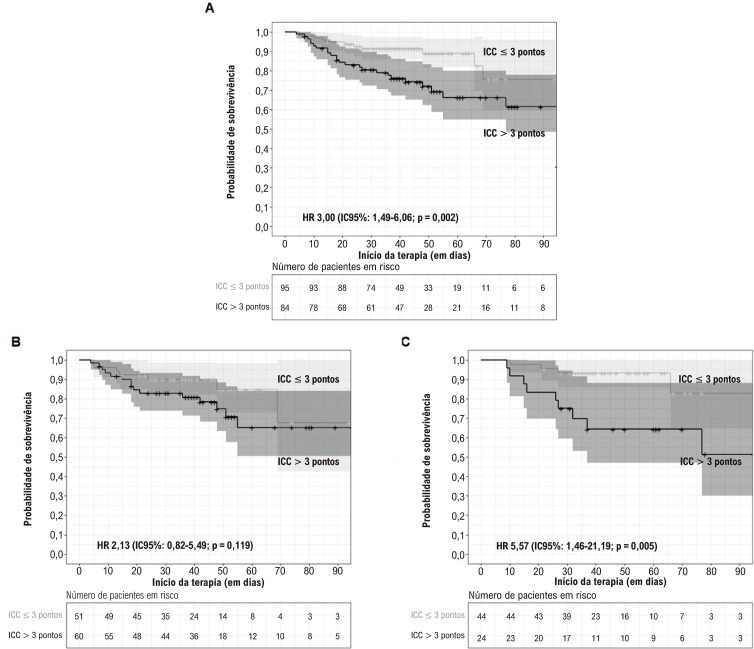
– Sobrevida hospitalar de acordo com o Índice de Comorbidade de Charlson (ICC) na amostra total (4A), (4B) pacientes não operados e (4C) pacientes operados; ICC: Índice de Comorbidade de Charlson comorbidity; IC: intervalo de confiança; HR: hazard ratio; o sombreamento representa o intervalo de confiança de 95% da estimativa.

Também encontramos uma associação entre ICC>3 pontos e menor sobrevida hospitalar em pacientes operados; no subgrupo de pacientes não operados, no entanto, não houve associação estatisticamente significativa entre ICC elevada e menor mortalidade hospitalar. Curvas de sobrevida hospitalar segundo o ICC estão apresentadas na Figura 4.

### Análise de sobrevida após a alta hospitalar

Dos 135 pacientes que receberam alta após a primeira internação por EI, 73 (54,1%) morreram durante o acompanhamento; 25 (34,2%) das mortes foram registradas no primeiro ano de seguimento. O tempo mediano (IIQ) de acompanhamento foi de 8,95 (3,23-14,1) anos, correspondendo a 1223 pacientes-anos e a uma taxa de incidência de seis eventos por 100 paciente-anos. A sobrevida após a alta foi 12,3 (± 3,30) anos. Não houve diferença estatisticamente significativa nas taxas de mortalidade após a alta entre os pacientes operados e não operados (58,5 vs. 47,2% respectivamente, p = 0,264).

**Figura 5:**
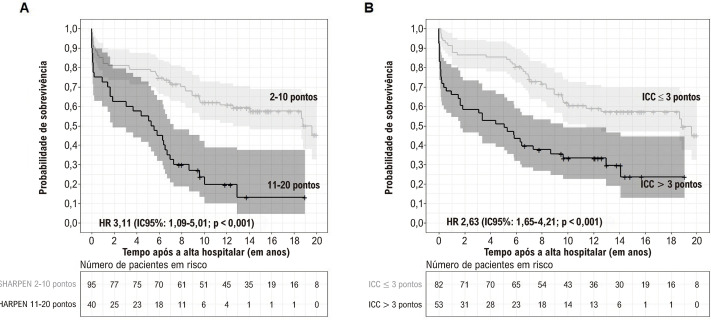
– Sobrevida após a alta de acordo com o escore SHARPEN (5A) e o Índice de Comorbidade de Charlson (5B); ICC: índice de comorbidade de Charlson; IC: intervalo de confiança; HR: hazard ratio; o sombreamento representa o intervalo de confiança de 95% da estimativa.

As taxas de mortalidade após a alta foram maiores nos pacientes com um escore SHARPEN de 11-20 pontos (80,0 vs. 43,2% 2-10 pontos; p < 0,001) e naqueles com um ICC > 3 pontos (69,8 vs. 43,9% ICC ≤ 3 pontos; p = 0,006). A taxa de sobrevivência foi mais baixa nos pacientes com um escore SHARPEN de 11-20 pontos e um ICC > 3 pontos. A Figura 5 apresenta as curvas de Kaplan-Meier para sobrevida após a alta de acordo com ambos os escores avaliados no estudo.

### Avaliação prognóstica usando o escore SHARPEN e o ICC para predição de mortalidade após a alta

**Figura 6:**
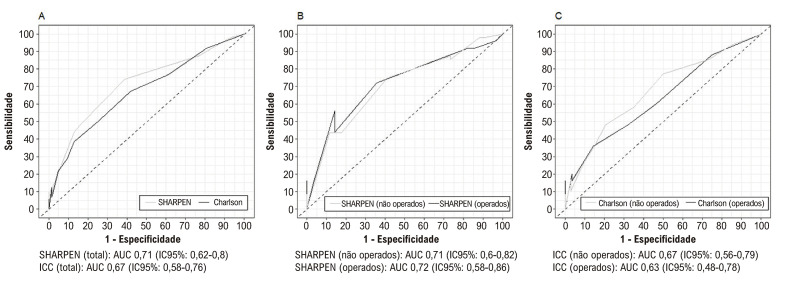
– Curvas do escore de SHARPEN e índice de comorbilidade de Charlson para predição de mortalidade após a alta. 6A: Curvas ROC para escore SHARPEN e índice de comorbilidade de Charlson na amostra total; 6B: Curvas ROC para escore SHARPEN para os pacientes operados e os pacientes não operados; 6C: Curvas ROC para índice de comorbilidade de Charlson para os pacientes operados e os pacientes não operados; ICC: índice de comorbilidade de Charlson; AUC: área sob a curva; IC: intervalo de confiança.

A Figura 6 apresenta as curvas ROC para predição de mortalidade após a alta de acordo com o escore SHARPEN e o ICC. Não houve diferença estatisticamente significativa nas AUCs do escore SHARPEN entre os pacientes operados e não operados (p = 0,086). Ainda, não houve diferença nas AUCs do ICC entre pacientes operados e não operados (p = 0,683). Não foram observadas diferenças entre o escore SHARPEN e o ICC na predição de mortalidade após a alta na população total (p = 0,515), nos pacientes operados (p = 0,547) e nos pacientes não operados (p = 0,468).

## Discussão

Nosso estudo apresentou três importantes conclusões: 1) o escore SHARPEN é preciso na predição de mortalidade hospitalar tanto em pacientes operados como não operados; 2) a acurácia do escore SHARPEN é similar à do ICC e significativamente melhor nos pacientes não operados; 3) Escores SHARPEN e ICC mais altos estão associados com mortalidade elevada após a alta de uma internação por EI.

As taxas de mortalidade na nossa amostra foram similares às descritas no estudo de Chee et al.^11^ (22,3 vs. 23,2%, respectivamente). Um estudo brasileiro do tipo coorte, publicado por Lemos et al., que incluiu 359 pacientes entre 2006 e 2019, 285 (79,4%) desses operados, mostrou uma mortalidade hospitalar de 24,5%, a qual também foi comparável aos nosso resultados.^31^ Contudo, embora o estudo original^11^ e o registro multicêntrico EURO-ENDO^6^ tenham mostrado mortalidade reduzida nos pacientes com EI que não tinham indicação de cirurgia, nós não encontramos diferença na mortalidade na comparação entre pacientes operados e não operados. Uma possível explicação para essas divergências é a existência de um viés de sobrevivência no qual pacientes elegíveis para cirurgia que foram operados têm maior probabilidade de sobreviver, enquanto aqueles com indicação cirúrgica que não puderam ser operados têm um prognóstico inerente pior.^11,32,33^ A taxa de cirurgia mais baixa no estudo original^11^ em comparação com a nossa (26,9 vs. 38,0%) sugere que a cirurgia teria sido contraindicada em pacientes em alto risco.^34^

O escore SHARPEN foi comparado ao ICC pela sua capacidade de predizer mortalidade hospitalar em estudos anteriores.^6,35,36^ apesar da acurácia relativamente baixa do ICC para predição de mortalidade hospitalar, um escore >3 foi associado com mortalidade elevada durante a internação, exceto no grupo não operado. Esse efeito pode estar relacionado à associação entre um ICC elevado e taxas de cirurgia mais baixas,^6^ o que também é um preditor independente de mortalidade.^36^ Similar ao estudo de Lu et al.,^37^ o ICC também foi capaz de predizer mortalidade em longo prazo na nossa amostra.

**Figura 3:**
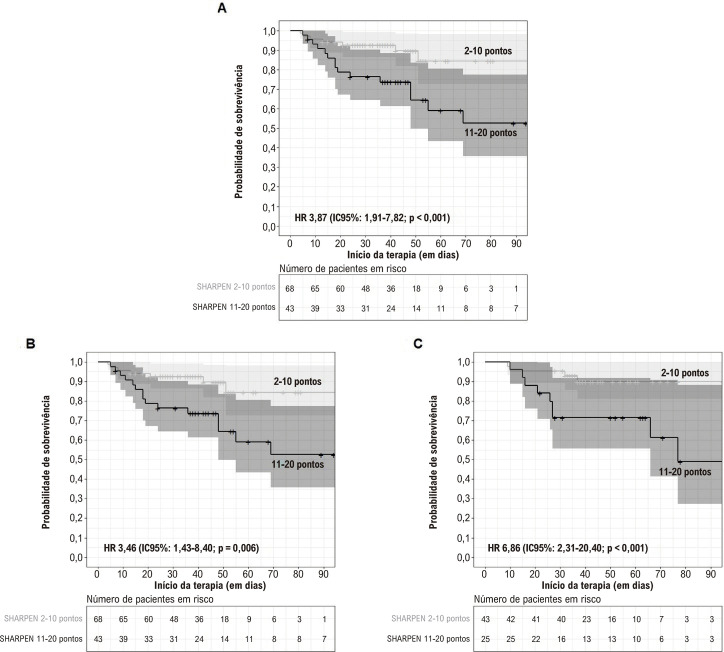
– Sobrevida hospitalar de acordo com o escore SHARPEN (3A) total (3B) pacientes não operados (3C) e operados. IC: intervalo de confiança; HR: hazard ratio; o sombreamento representa 95% da estimativa.

A aplicação dos escores de risco desenvolvidos especificamente para paciente com EI é preferível devido às particularidades da doença que nem sempre são contempladas por escores de risco de uso geral. Essa crença é reforçada pelo fato de que o EuroSCORE, um dos principais escores de risco cirúrgico usados na prática clínica, subestimou a mortalidade em cirurgia de válvula para EI ativa.^38^ Embora seja muito interessante ter escores específicos para a avaliação da mortalidade na EI, vale a pena ressaltar que o escore SHARPEN também inclui aspectos gerais (por exemplo, uso de drogas vasoativas para IC, presença de insuficiência renal, pressão arterial) que são, na verdade, incluídos em outros escores como o SOFA e o qSOFA.^39,40^

Poucos estudos abordaram a aplicação dos escores de risco cirúrgico em pacientes com EI não operados. Gatti et al.^41^ relataram que três escores específicos para EI (STS-IE, escore ICE, e EndoSCORE) e dois escores específicos para cirurgia cardíaca (logistic EuroSCORE e EuroSCORE II) tiveram um desempenho satisfatório. Contudo, a avaliação prognóstica pode ser enviesada por vários fatores: escores cirúrgicos contêm variáveis relacionadas ao risco perioperatório, que são de pouca relevância a pacientes não operados. Além disso, a omissão dos pacientes não operados do processo de validação aumenta o risco de viés de sobrevivência.^32,33^ Considerando que o escore SHARPEN foi desenvolvido e validado especificamente para pacientes com EI independentemente da necessidade de cirurgia e que mostrou melhor poder discriminatório em pacientes não operados, sua aplicação poderia ser mais vantajosa.

Este é o primeiro estudo a analisar o desempenho do escore SHARPEN na predição de mortalidade em longo prazo. Embora sua acurácia tenha sido considerada baixa, pacientes com um escore elevado têm taxas baixas de sobrevivência, mesmo tendo completado o tratamento para EI. IC e idade, ambos componentes do escore SHARPEN, também foram preditores independentes de mortalidade após a alta no estudo de Tahon et al.^42^ Devido ao tamanho reduzido da nossa amostra, não conseguimos avaliar o desempenho do SHARPEN nos pacientes operados e não operados separadamente.

O presente estudo tem limitações. A amostra foi relativamente pequena e restrita à um único centro terciário. A baixa média de 10,5 pacientes com EI/ano também pode ser considerada uma limitação. No longo período de análise, tanto o manejo clínico como o cirúrgico desses pacientes pode ter mudado ao longo do tempo. A coleta retrospectiva de dados pode comprometer a qualidade dos dados obtidos. Finalmente, o número de pacientes em risco reduz à medida que o tempo após a alta hospitalar aumenta, o que reduz a validade dos dados (como pode ser visto a partir dos IC95% nas curvas de sobrevida).

Apesar da necessidade de estudos multicêntricos maiores, a acurácia aceitável e o alto valor preditivo negativo do escore SHARPEN em nossa amostra sugerem que ele pode ser útil na prática clínica para selecionar pacientes em alto risco que requerem cuidado otimizado durante a internação e um acompanhamento de perto após a alta para prevenir desfechos adversos. Embora o escore SHARPEN seja composto por variáveis que são facilmente obtidas, o escore de risco foi desenvolvido para ser calculado logo após o diagnóstico de EI, que pode ocorrer em estágios variados da internação. Como uma perspectiva futura, propomos a análise do valor prognóstico da reclassificação do paciente durante a internação.

## Conclusão

O escore SHARPEN foi reprodutível como um preditor de mortalidade hospitalar tanto em pacientes operados como não operados, com uma acurácia aceitável. Além disso, verificamos que os pacientes classificados como alto risco permaneceram com uma mortalidade significativamente maior alta após a alta em comparação a pacientes com baixo risco. Embora a acurácia do escore SHARPEN para a predição de mortalidade hospitalar tenha sido similar à do ICC na população toal, observou-se uma acurácia significativamente maior nos pacientes não operados. Assim, nossos achados destacam os potenciais benefícios da aplicação do escore SHARPEN na prática clínica.

## Footnotes

* Material suplementar Para informação adicional, por favor, clique aqui.
