# Supplementary material for: Desempenho do Escore SHARPEN e do Índice de Comorbidade de Charlson para Predição de Mortalidade durante a Internação Hospitalar e após a Alta na Endocardite Infecciosa
Source: Arq Bras Cardiol. 2024 Jan 19;120(12):e20230441. [Article in Portuguese] doi: 10.36660/abc.20230441 (PMC11021123; doi:10.36660/abc.20230441)
Supplement: Translation

# Translation: Performance of the SHARPEN Score and the Charlson Comorbidity Index for In-Hospital and Post-Discharge Mortality Prediction in Infective Endocarditis

**Authors:** Sofia Giusti Alves, Fernando Pivatto, Filippe Barcellos Filippini, Gustavo Paglioli Dannenhauer, Gabriel Seroiska, Helena Marcon Bischoff, Luiz Felipe Schmidt Birk, Diego Henrique Terra, Daniel Sganzerla, Marcelo Haertel Miglioranza

**Affiliations:** 1 Brazil Hospital de Clínicas de Porto Alegre (HCPA), Porto Alegre, RS – Brazil; 2 Brazil Hospital Nossa Senhora da Conceição (HNSC), Porto Alegre, RS – Brazil; 3 Brazil Instituto de Cardiologia de Santa Catarina, São José, SC – Brazil; 4 Brazil Universidade Federal de Ciências da Saúde de Porto Alegre (UFCSPA), Porto Alegre, RS – Brazil; 5 Brazil Unimed Porto Alegre Cooperativa Médica, Porto Alegre, RS – Brazil; 6 Brazil Instituto de Cardiologia do Rio Grande do Sul (ICFUC-RS) – Laboratório de Pesquisa e Inovação em Imagem Cardiovascular, Porto Alegre, RS – Brazil; 7 Brazil Hospital Mãe de Deus, Porto Alegre, RS – Brazil

**Keywords:** Keywords: Endocarditis, Comorbidity, Survival Analysis

## Abstract

**Background:**

SHARPEN was the first dedicated score for in-hospital mortality prediction in infective endocarditis (IE) regardless of cardiac surgery.

**Objectives:**

To analyze the ability of the SHARPEN score to predict in-hospital and post-discharge mortality and compare it with that of the Charlson comorbidity index (CCI).

**Methods:**

Retrospective cohort study including definite IE (Duke modified criteria) admissions from 2000 to 2016. The area under the ROC curve (AUC-ROC) was calculated to assess predictive ability. Kaplan-Meier curves and Cox regression was performed. P-value < 0.05 was considered statistically significant.

**Results:**

We studied 179 hospital admissions. In-hospital mortality was 22.3%; 68 (38.0%) had cardiac surgery. Median (interquartile range, IQR) SHARPEN and CCI scores were 9(7-11) and 3(2-6), respectively. SHARPEN had better in-hospital mortality prediction than CCI in non-operated patients (AUC-ROC 0.77 vs. 0.62, p = 0.003); there was no difference in overall (p = 0.26) and in operated patients (p = 0.41). SHARPEN > 10 at admission was associated with decreased in-hospital survival in the overall (HR 3.87; p < 0.001), in non-operated (HR 3.46; p = 0.006) and operated (HR 6.86; p < 0.001) patients. CCI > 3 at admission was associated with worse in-hospital survival in the overall (HR 3.0; p = 0.002), and in operated patients (HR 5.57; p = 0.005), but not in non-operated patients (HR 2.13; p = 0.119). Post-discharge survival was worse in patients with SHARPEN > 10 (HR 3.11; p < 0.001) and CCI > 3 (HR 2.63; p < 0.001) at admission; however, there was no difference in predictive ability between these groups.

**Conclusion:**

SHARPEN was superior to CCI in predicting in-hospital mortality in non-operated patients. There was no difference between the scores regarding post-discharge mortality.

## Introduction

Infective endocarditis (IE) has a high incidence, with 1.5-11.6 cases per 100,000 population,^1^ and in-hospital mortality rates ranging from 17.5 to 30%.^1-3^ Patients who survive the first episode of IE continue to present with excess mortality and morbidity, especially within the first year after discharge.^4^ Recent changes in IE epidemiological profile may have contributed to the maintenance of elevated morbidity and mortality. IE incidence has increased in patients with risk factors for adverse outcomes, such as advanced age, multiple comorbidities, prosthetic valves, and intracardiac devices; also, an increase in nosocomial cases has been reported.^2,5,6^

Considering IE a significant health burden, optimizing evaluation and treatment is essential. There is evidence of improvement in clinical outcomes with the use of a multidisciplinary alert strategy and standardized IE protocols based on disease severity.^7-10^ In this context, score-based risk stratification can refer high-risk patients to specialized or intensive care.^11^ Considering that early surgery has been shown to reduce IE mortality, risk scores may be particularly useful to guide patient selection.^12^ Furthermore, its application for mortality prediction after a hospitalization due to IE could help identify patients who would benefit from a closer post-discharge follow-up.

Several surgical risk scores have been developed specifically for IE^13-20^ and compared with traditional surgical risk scores, such as EuroSCORE and the STS score.^21-23^ However, these scores have not been validated in non-operated patients who represent almost 50% of all hospitalizations for IE^6^ or for long-term prognostic evaluation. The ICE-Prospective Cohort Study is an exception regarding evaluation of mortality for IE after discharge, which was determined at six months.^24^ Therefore, it is necessary to improve the prognostic evaluation of patients undergoing medical therapy alone.

SHARPEN is a risk score for IE developed by Chee et al.^11^ to predict in-hospital mortality in operated and non-operated patients.^25^ Our study aims to prognostic value of SHARPEN score during hospitalization and after discharge in both operated and non-operated patients admitted for IE and to compare its performance with that of the Charlson comorbidity index (CCI).^26^

## Patients and methods

We performed a unicentric retrospective cohort study enrolling all active IE cases (on antibiotic treatment)^27^ from 2000 to 2016 in patients aged ≥ 18 years. Only patients with a definite diagnosis of IE according to the modified Duke criteria were included.^28^ Our institution is a public tertiary teaching hospital located in southern Brazil. It has 784 beds and access to care is provided exclusively through the Brazilian Unified Health System, mostly to low-income patients. An average of 30-60 valve replacements are performed per year at the institution. The study was approved by the institution Research Ethics Committee.

Hospitalizations for IE were identified by the International Classification of Diseases, 10^th^ revision (ICD-10) code^29^recorded in the discharge summary or at any point during hospitalization. The following codes were searched: B37.6 (Candidal endocarditis), I33.0 (acute and subacute IE), I33.9 (acute and subacute endocarditis, unspecified), I38 (endocarditis, valve unspecified), and I39.8 (endocarditis and heart valve disorders in diseases classified elsewhere). After this initial screening, the patients’ medical records were reviewed to ensure that inclusion criteria were met. Figure 1 shows the study flowchart. Data regarding the hospitalization period were collected from electronic and physical medical records. Post-discharge follow-up assessment involved evaluation of medical charts to verify whether the surviving patients had appointments and/or hospitalizations after hospital discharge, telephone number of the remaining patients and, finally, review of death certificates. All patients that were not registered as deceased in neither of these data sources by October 1^st^, 2022 (which marked the conclusion of the follow-up assessment) were considered alive.

**Figure 1:** – Flowchart. IE: infective endocarditis; ICD-10: International Classification of Diseases, 10th revision; CCI: Charlson comorbidity index. *Modified Duke criteria. Data presented as n (%).

SHARPEN score^11^ (2-20 points, Supplementary File S1) was calculated retrospectively for each hospitalization which was classified as low or high risk for in-hospital mortality according to the best cut-off point observed. Three points were assigned to systolic blood pressure < 90 mm Hg at presentation or non-intravenous (IV) drug abuser; two points to manifestations of heart failure (HF) during hospitalization, creatinine at admission > 2.26 mg/dL, diagnosis of nosocomial pneumonia, or peak C-reactive protein (CRP) > 200 mg/dL during hospitalization; and 2, 4, and 6 points to age groups < 50, 50-65, and > 65 years, respectively. The diagnosis of HF was defined based on the Framingham criteria.^30^ Nosocomial pneumonia was defined as pneumonia occurring ≥ 48 hours after hospital admission. The CCI was calculated for each patient (Supplementary File S2), and the comorbidity definitions of the original study were used.^26^ The urgency of operation was defined according to the EuroSCORE II criteria.^27^

### Statistical analysis

Data were analyzed using SPSS, version 21.0, and MedCalc, version 12.5. For descriptive analysis, categorical variables were expressed as absolute and relative frequencies, continuous variables with normal distribution as mean (± standard deviation [SD]), and continuous variables with non-normal distribution as median (interquartile range [IQR]). The Shapiro-Wilk test was used to test the normality of distribution. For between-group comparisons, categorical variables were compared by the chi-square test and quantitative variables were compared by Student’s t test if normally distributed or by the Mann-Whitney U test if not normally distributed. Fisher exact test was used in case of low data frequency. The predictive ability of SHARPEN score and CCI was assessed by calculating the area under the ROC curve (AUC-ROC). AUC-ROCs were compared using the DeLong test, and the best cut-off point in each scoring system was determined by the Youden index. Survival analysis was performed using Kaplan-Meier curves. Cox regression models were used to calculate the hazard ratio (HR) of in-hospital and post-discharge mortality. P-value < 0.05 was considered statistically significant.

## Results

### Baseline characteristics of the sample

Of 251 hospitalizations initially identified, 179 hospital admissions of 168 patients met the inclusion criteria; 10 patients had more than one admission for active IE during the study period (Supplementary File S3). The median (IQR) length of hospital stay was 45 (33-64) days, with 93 (52.0%) admission to the intensive care unit (excluding admissions for postoperative monitoring only). The baseline characteristics of the sample are shown in Table 1. Antibiotic therapy was initiated at a median of 1 (0-6) day after hospital admission. There were no cases of IE in cardiac devices.

**Table 1:** – Baseline characteristics and in-hospital complications

Variable	Overall (n = 179)	Hospital discharge (n = 139)	In-hospital death (n = 40)	p
***Baseline characteristics***				
Age (years)*	57.4 (42.3-68.5)	54.7 (40.6-66.0)	64.7 (54.5-72.1)	.004
Male	126 (70.4)	101 (72.7)	25 (62.5)	.30
LVEF (%)	63 (58-68)	63 (58-68)	62 (56-69)	.97
Left-sided IE	164 (91.6)	124 (89.2)	40 (100)	.020
Hypertension	92 (51.4)	68 (48.9)	24 (60.0)	.29
Diabetes†	37 (20.7)	27 (19.4)	10 (25.0)	.58
Previous cardiac surgery	31 (17.3)	24 (17.3)	7 (17.5)	1.0
Systolic BP < 90mmHg at presentation*	26 (14.5)	15 (10.9)	11 (27.5)	.018
Peak CRP during hospitalization > 200mg/L*	26 (14.5)	16 (11.5)	10 (25.0)	.060
Prosthetic valve IE	23 (12.8)	18 (12.9)	5 (12.5)	1.0
Creatinine > 2.26mg/dL at presentation*	20 (11.2)	12 (8.6)	8 (20.0)	.082
CKD requiring dialysis†	14 (7.8)	8 (5.8)	6 (15.0)	.088
Intravenous drug abuse*	13 (7.3)	11 (7.9)	2 (5.0)	.74
HIV	9 (5.0)	6 (4.3)	3 (7.5)	.42
Cardiac dysfunction (LVEF ≤ 40%)	8 (4.5)	6 (4.3)	2 (5.0)	.85
***In-hospital complications***				
Moderate/severe regurgitation	107 (59.8)	79 (56.8)	28 (70.0)	.19
Heart failure*†	97 (54.2)	69 (49.6)	28 (70.0)	.036
Cardiac surgery	68 (38.0)	54 (38.8)	14 (35.0)	.80
Pneumonia (≥ 48h after admission)*	38 (21.2)	22 (15.8)	16 (40.0)	.002
Embolic events (excluding cerebrovascular events)	35 (19.6)	25 (18.0)	10 (25.0)	.45
Hemodialysis‡	33 (18.4)	12 (9.2)	21 (61.8)	< .001
Intracranial complications (hemorrhage/ischemic stroke)	31 (17.3)	21 (15.1)	10 (25.0)	.22
Ruptured chordae tendineae	23 (12.8)	19 (13.7)	4 (10.0)	.73
Perivalvular abscess	18 (10.1)	14 (10.1)	4 (10.0)	1.0
Fistula	14 (7.8)	10 (7.2)	4 (10.0)	.52
Pseudoaneurysm	3 (1.7)	3 (2.2)	0 (0)	1.0

Data presented as median (interquartile range) or number (%). CKD: Chronic kidney disease; HIV: human immunodeficiency virus; IE: infective endocarditis; LVEF: left ventricular ejection fraction; NS: non-significant; BP: blood pressure.*Component of the SHARPEN score. †Component of the Charlson comorbidity index.‡Excluding patients with pre-admission chronic kidney disease requiring dialysis (n = 14).

Transesophageal echocardiography was performed in most patients (n = 145; 81.0%). In 162 (90.5%) patients, vegetations were detected, which were larger than 10 mm in 69 (38.5%) of them. Aortic (n = 68; 38.0%) and mitral valve (n = 60; 33.5%) IE were the most common presentations, and 36 (20.1%) patients had more than one valve involved. Positive blood cultures were found in 154 (86.0%) patients; *Staphylococcus aureus* (22.0%) and viridans group streptococci (15.1%) were the most common agents. Intravenous catheter-associated infection occurred in 12 (6.7%) of the sample. There were no cases of intracardiac devices infection.

While 87 (48.6%) hospitalizations had surgical indication, the procedure was performed during the same admission in only 68 (78.2%) of them - urgently in 64 (94.1%) and on an emergency basis in 4 (5.9%) patients. The most common surgical indications were acute HF (n = 54; 79.4%) and uncontrolled infection (n = 28; 41.2%). The main reasons for not operating at the same hospitalization despite indication were the following: planning to undergo elective surgery in a subsequent hospitalization (n = 7; 6.8%) and having hemodynamic instability (n = 5; 26.3%). Mechanical aortic valve replacement (n = 19, 10.6%) and biological mitral valve replacement (n = 12, 6.7%) were the most frequently performed procedures. The mean (± SD) times of cardiopulmonary bypass and ischemia were 136 (± 46) and 104 (± 42) minutes, respectively.

In-hospital mortality was 22.3% (95% confidence interval [CI]: 16.2-28.4%), and septic shock was the main cause of death (n = 20; 11.2%). Mortality was similar in operated and non-operated patients (20.6 vs. 23.4%, p = 0.797).

**Figure 3:** – In-hospital survival according to SHARPEN score in (3A) overall, (3B) non-operated and (3C) operated patients. CI: confidence interval; HR: hazard ratio. The line shadow represents the 95% confidence interval of the estimate.

### Prognostic evaluation using the SHARPEN score and the CCI for in-hospital mortality prediction

Patients had a median (IQR) SHARPEN score of 9 (7-11) points. Median (IQR) SHARPEN scores of patients discharged from the hospital and those who died during hospitalization were 9 (7-11) and 11 (9-13) points, respectively (p < 0.001). The best cut-off point observed for mortality prediction in the SHARPEN score was > 10 points. Overall, 111 (62.0%) hospitalizations were classified as low (2-10 points) and 68 (38.0%) as high risk (11-20 points) with in-hospital mortality rates of 10.8 and 41.2%, respectively (p < 0.001).

Patients had a median (IQR) CCI of 3 (2-6) points. Median (IQR) CCI of patients discharged from the hospital and those who died during hospitalization were 3 (1-5) and 5 (3-7), respectively (p < 0.001). The optimal cut-off point for mortality prediction in CCI was > 3 points. Overall, 95 (56.1%) hospitalizations were classified as low (≤ 3 points) and 84 (46.9%) as high risk (> 3 points) with in-hospital mortality rates of 12.5 and 33.3%, respectively (p < 0.001). Table 2 shows the characteristics of the SHARPEN score and the CCI in overall, non-operated and operated patients.

**Table 2:** – Characteristics of the SHARPEN score and Charlson comorbidity index

Statistics % (95%CI)	SHARPEN > 10			CCI > 3		
	Overall	Non-operated	Operated	Overall	Non-operated	Operated
Sensitivity	70.0 (53.5-83.4)	71.4 (41.9-91.1)	69.2 (48.2-85.7)	70.0 (53.5-83.4)	69.2 (48.2-85.7)	71.4 (41.9-91.6)
Specificity	71.2 (62.9-78.6)	72.2 (58.3-83.5)	70.6 (59.7-79.9)	59.7 (51.1-67.9)	50.6 (39.5-61.6)	74.1 (60.4-85)
Positive likelihood ratio	2.43 (1.75-3.39)	2.57 (1.49-4.43)	2.35 (1.55-3.57)	1.74 (1.30-2.31)	1.40 (1.0-1.96)	2.76 (1.57-4.82)
Negative likelihood ratio	0.42 (0.26-0.68)	0.40 (0.17-0.92)	0.44 (0.24-0.79)	0.50 (0.31-0.82)	0.61 (0.33-1.12)	0.39 (0.17-0.90)
Mortality	22.3	20.6	23.4	22.3	23.4	20.6
Positive predictive value	41.1 (33.4-49.3)	40.0 (27.9-53.4)	41.8 (32.1-52.2)	33.3 (27.2-39.9)	30 (23.5-37.5)	41.67 (28.9-55.5)
Negative predictive value	89.2 (83.6-93.1)	90.7 (80.7-95.8)	88.2 (80.6-93.1)	87.4 (80.9-91.9)	84.3 (74.4-90.9)	90.9 (81.1-95.9)
Accuracy	70.9 (63.7-77.5)	72.1 (59.8-82.3)	70.3 (60.8-78.6)	62.0 (54.5-69.1)	54.95 (45.2-64.4)	73.5 (61.4-83.5)

*CCI: Charlson comorbidity index; CI: confidence interval.*

Figure 2 shows the ROC curves for in-hospital mortality prediction according to the SHARPEN and the CCI. There was no difference in the AUC of the SHARPEN score between operated and non-operated patients (p = 0.058). On the other hand, we found a statistically significant difference in the AUC of the CCI between operated and non-operated patients (p = 0.039). When comparing the SHARPEN and CCI abilities to predict in-hospital mortality, we found no difference in the overall sample (p = 0.26) and in operated patients (p = 0.41). However, in the non-operated subgroup, the SHARPEN score was superior to the CCI (p = 0.003).

**Figure 2:** – SHARPEN score and Charlson Comorbidity Index’s ROC curves for in-hospital mortality prediction. 2A: ROC curves for SHARPEN score and Charlson Comorbidity Index in the whole sample. 2B: ROC curves for SHARPEN score in operated and non-operated patients. 2C: ROC curves for Charlson Comorbidity Index in operated and non-operated patients. CCI: Charlson comorbidity index; AUC: area under the curve; CI: confidence interval.

### In-hospital survival analysis

There is a statistically significant association between SHARPEN score > 10 and decreased in-hospital survival, which persisted when analyzing the non-operated and operated patients separately. In-hospital survival curves according to the SHARPEN score are shown in Figure 2.

We also found an association between CCI > 3 points and decreased in-hospital survival in overall and in operated patients; in the non-operated subgroup, however, there was no statistically significantly association between an elevated CCI and decreased in-hospital mortality. In-hospital survival curves according to the CCI are shown in Figure 4.

**Figure 4:** – In-hospital survival according Charlson comorbidity index (CCI) in (4A) overall, (4B) non-operated and (4C) operated patients. CCI: Charlson comorbidity index; CI: confidence interval; HR: hazard ratio. The line shadow represents the 95% confidence interval of the estimate.

### Post-discharge survival analysis

Out of the 135 patients that received hospital discharge after the first hospitalization due to IE, 73 (54.1%) died during the follow-up; 25 (34.2%) of the deaths were registered in the first year of follow-up. The median (IQR) post-discharge follow-up was 8.95 (3.23-14.1) years, which corresponded to 1,223 patient-years and an incident rate of six events per 100 patient-years. The mean (± SD) post-discharge survival was 12.3 (± 3.30) years. There was no statistically significant difference in post-discharge mortality rates between non-operated and operated patients (58.5 vs. 47.2% respectively, p = 0.264).

Post-discharge death rates were significantly higher in patients with a SHARPEN score of 11-20 points (80.0 vs. 43.2% 2-10 points; p < 0.001) and in those with a CCI > 3 points (69.8 vs. 43.9% CCI ≤ 3 points; p = 0.006). Survival was lower in patients with a SHARPEN score 11-20 points and CCI > 3 points. Figure 5 shows the Kaplan-Meier curves for post-discharge survival according to both scores evaluated in our study.

**Figure 5:** – Post-discharge survival according to (5A) SHARPEN score and (5B) Charlson comorbidity index. CCI: Charlson comorbidity index; CI: confidence interval; HR: hazard ratio. The line shadow represents the 95% confidence interval of the estimate.

### Prognostic evaluation using the SHARPEN score and the CCI for post-discharge mortality prediction

Figure 6 shows the ROC curves for post-discharge mortality prediction according the SHARPEN and the CCI. There was no statistically significant difference in the AUCs of the SHARPEN score between operated and non-operated patients (p = 0.086). Also, there was no difference in the AUCs of the CCI between operated and non-operated patients (p = 0.683). No differences were found between the SHARPEN score and the CCI to predict post-discharge mortality, in the overall (p = 0.515), operated (p = 0.547) and non-operated groups (p = 0.468).

**Figure 6:** – SHARPEN score and Charlson Comorbidity Index’s ROC curves for post-discharge mortality prediction; 6A: ROC curves for SHARPEN score and Charlson Comorbidity Index in the overall sample; 6B: ROC curves for SHARPEN score in operated and non-operated patients; 6C: ROC curves for Charlson Comorbidity Index in operated and non-operated patients. CCI: Charlson comorbidity index; AUC: area under the curve; CI: confidence interval.

## Discussion

Results of our study revealed three important findings: 1) SHARPEN score is accurate in predicting in-hospital mortality in both operated and non-operated patients; 2) the accuracy of the SHARPEN score is similar to that of the CCI and significantly better in non-operated patients; 3) Higher SHARPEN and CCI scores are associated with elevated mortality after discharge from a hospitalization due to IE.

Hospital mortality rates in our sample were similar to those described in the study by Chee et al.^11^ (22.3 vs. 23.2%, respectively). A Brazilian cohort study published by Lemos et al., including 359 patients from 2006 to 2019, 285 (79.4%) operated, showed an in-hospital mortality of 24.5%, which is also comparable to ours.^31^However, even though both the original study^11^ and the multicentric EURO-ENDO registry^6^ have shown reduced mortality in IE patients who did not have an indication for surgery, we found no difference in mortality when comparing operated and non-operated patients. A possible explanation for these divergences is the existence of a “survivor bias”, in which patients who are fit for surgery are more likely to survive, while those unable to undergo surgery despite indications have an inherently worse prognosis.^11,32,33^ The lower surgical rate in the original study,^11^ when compared to ours (26.9 vs. 38.0%), suggests that surgery may have been contraindicated in high-risk patients.^34^

The SHARPEN score was compared with the CCI due to its ability to predict in-hospital mortality in previous studies.^6,35,36^ Even though the CCI had a relatively poor accuracy for in-hospital mortality prediction, a score > 3 was associated with elevated mortality during the hospitalization, except in the non-operated group. This effect may be related to the association between an elevated CCI and lower surgery rates,^6^ which was also found to be an independent predictor of mortality.^36^ Similarly to the study by Lu et al.,^37^ the CCI was also able to predict long-term mortality in our sample.

The application of risk scores specifically designed for IE patients is preferable due to the particularities of this disease, which are not always contemplated by general-use risk scores. This belief is reinforced by the fact that the EuroSCORE, one of the main surgical risk scores used in clinical practice, was shown to underestimate mortality in valvular surgery for active IE.^38^ Although it is very interesting to have specific scores for assessment of mortality in IE, it is worth highlighting that the SHARPEN score also includes general aspects (e.g. use of vasoactive drugs for HF, presence of renal failure, blood pressure), that are in fact included in other scores like the SOFA^39^ and qSOFA^40^ scores.

Few studies have addressed the application of surgical risk scores in non-operated patients with IE. Gatti et al.^41^reported that three scores specific for IE (STS-IE, ICE score, and EndoSCORE) and two scores specific for cardiac surgery (logistic EuroSCORE and EuroSCORE II) had a satisfactory performance. However, the prognostic assessment may be biased by several factors: surgical scores contain variables related to perioperative risk, which are of little relevance to non-operated patients; besides, the omission of non-operated patients from the validation process increases the risk of survivor bias.^32,33^ Considering that the SHARPEN score was designed and validated specifically for IE patients, regardless of the need for surgery, and showed improved discriminatory power in non-operated patients, its application would be more advantageous.

This is the first study to analyze the SHARPEN score performance in long-term mortality prediction. Even though its accuracy was considered poor, patients with an elevated score have lower survival rates after discharge despite having completed treatment for IE. HF and age, both of them components of the SHARPEN score, were also found to be independent predictors of mortality after hospital discharge in a study by Tahon et al.^42^ Due to the reduced size of our sample, we were not able to evaluate the SHARPEN performance separately in operated and non-operated groups.

The present study has limitations. The sample was relatively small and restricted to a single tertiary care center. The low mean of 10.5 IE patient/year can also be considered a limitation. In this long period of analysis, both clinical and surgical management of these patients may have changed over time. Retrospective data collection can compromise the quality of the data obtained. Finally, the number of patients at risk greatly reduces with increasing time after hospital discharge, which reduces the validity of the data (as can be seen from the 95%CIs in the survival curves).

Despite the need for larger multicenter studies, the acceptable accuracy and high negative predictive value of the SHARPEN score in our sample suggest that it may be useful in clinical practice to select high-risk patients that require optimized care during hospitalization and close follow-up after discharge in order to prevent adverse outcomes. Although the SHARPEN score is composed of easily obtainable variables, this risk score was designed for calculation right after the diagnosis of IE, which may occur in varying stages of the hospitalization. As a future perspective, we propose the analysis of the prognostic value of patient reclassification during hospitalization.

## Conclusion

The SHARPEN score was reproducible as a predictor of in-hospital mortality in both operated and non-operated IE patients with an acceptable accuracy. Furthermore, we found that patients classified as high-risk persisted with a significantly higher mortality after hospital discharge as compared with low-risk patients. Although the SHARPEN score accuracy to predict in-hospital mortality was similar to that of the CCI in the overall EI patients, there was a significantly better accuracy in non-operated patients. Therefore, our findings highlight the potential benefits of applying the SHARPEN score in clinical practice.

## Footnotes

* Supplemental Materials For additional information, please click here.
